# Intra-Individual Variability Across Fluid Cognition Can Reveal Qualitatively Different Cognitive Styles of the Aging Brain

**DOI:** 10.3389/fpsyg.2018.01973

**Published:** 2018-10-16

**Authors:** Sara De Felice, Carol A. Holland

**Affiliations:** ^1^Institute of Neurology, Faculty of Brain Sciences, University College London, London, United Kingdom; ^2^Division of Health Research, Centre for Ageing Research (C4AR), Faculty of Health and Medicine, Lancaster University, Bailrigg, United Kingdom

**Keywords:** intra-individual variability, dispersion, fluid cognition, executive functions, aging, compensation, cognitive reserve, education

## Abstract

Dispersion is a measure of intra-individual variability reflecting how much performance across distinct cognitive functions varies within an individual. In cognitive aging studies, results are inconsistent: some studies report an increase in dispersion with increasing age and decline in performance, while others report an increasingly homogenous cognitive profile in older adults. We propose that inconsistencies may reflect qualitative differences in the cognitive functioning of the aging brain: age-groups may differ in how efficiently they engage resources, depending on both executive processing and resources available. This in turn would result in either greater or less dispersion. 21 young (mean 25.14 years, *SD* ± 2.85), 21 middle-old (65.05 ± 4.19), and 20 old-old (80.65 ± 4.38) healthy adults completed a series of neuropsychological tasks engaging executive processing, including switching, planning, updating, working memory and short-term memory. Individual dispersion profiles were obtained using a regression method which computes individual standard deviation across tasks from standardized test scores. Results revealed associations between performance, dispersion and cognitive reserve (measured as education level). Although differences across groups did not approach significance, there was a general pattern consistent with existing literature showing greater dispersion in the old-old group, and this was negatively associated with performance. In contrast, the middle-old group showed young-equivalent dispersion index, while performance was similar to the young group on some tasks and to the old-old group on others, possibly reflecting differences in cognitive demand. Educational level positively correlated with performance in the middle-old group only. Overall, a distinct pattern emerged for the middle-old adults: they showed young-equivalent performance on a number of measures and similar dispersion index, while uniquely benefitting from cognitive reserve. This may possibly reflect engagement in compensatory mechanisms. This study contributes to clarifying inconsistencies in previous studies and calls for more thoughtful selection of sample cohorts in aging research. The study of dispersion may provide a behavioral index of age-related changes in how cognition functions and recruits resources. Future work could examine whether this also reflects age-related changes in neural recruitment and aim at identifying factors contributing to cognitive reserve, in order to prolong good performance and improve cognition in aging.

## Introduction

The term intra-individual variability has been adopted to refer to variability in performance within individuals across either different trials (within the same task) or across tasks. In recent years, advanced aging has been studied with reference to intra-individual variability and pattern of performance within and across cognitive domains. While many studies have focused on intra-individual variability across trials within tasks (for a review see Dykiert et al., 2012), relatively less attention has been devoted to the study of intra-individual variability across tasks/cognitive functions, referred to in the literature as either dispersion (Hilborn et al., 2009) or differentiation (Juan-Espinosa et al., 2002; Li et al., 2004; Blair, 2006). This latter form of intra-individual variability is the focus of this study. While the terms intra-individual variability, differentiation and dispersion have been used interchangeably in the literature, the term “de-differentiation” specifically refers to reduced intra-individual variability within person across tasks (Balinsky, 1941; Juan-Espinosa et al., 2002). For clarity, here we will adopt the term ‘dispersion’ (or dispersion index) to refer to the variability in individuals’ performance across tasks, as defined in previous studies (Sosnoff and Newell, 2006; Hilborn et al., 2009; Halliday et al., 2018).

Reduced dispersion (cognitive de-differentiation) has been reported as a function of age across measures of speed of processing, working-memory, verbal fluency and lexical decision both in cross-sectional and longitudinal studies (Li et al., 2004; Rabbitt et al., 2004). However, more recent studies have reported the opposite pattern, namely an increase in dispersion with increasing age (Hultsch et al., 2000; Sosnoff and Newell, 2006; Hilborn et al., 2009; Halliday et al., 2018). These inconsistent results may derive from (i) differences in neuropsychological batteries, (ii) analysis adopted to compute dispersion index, and/or (iii) demographic differences in age groups across studies. Therefore, the present study will (i) use tests to include a variety of cognitive measures, (ii) compute dispersion index via a regression technique which accounts for external and internal confounds (see methods), and (iii) compare two different aged older adults groups with young adults. An alternative explanation may arise when considering the dispersion index in relation to cognitive performance across different older age groups, as this may carry information about the cognitive profile of a given age group, how cognition functions and recruits resources at different developmental stages. Specifically, the aim of this study is therefore to investigate variability in performance across cognitive measures within single individuals (dispersion index), and in particular its relation to cognitive performance in healthy aging.

Theoretical and empirical evidence points at the frontal lobe as the hub for neuronal and cognitive mechanisms that may be responsible for driving this cross-domain variability between individuals and age groups (e.g., Robbins et al., 1998; Rabbitt and Lowe, 2000; Gruber and Goschke, 2004; MacDonald et al., 2006). “Executive functions” is an umbrella term that refers to a series of cognitive processes which co-operate purposefully, including selecting the target information and inhibiting irrelevant information likely to interfere with current mental processes and/or response execution, keeping and manipulating information online, shifting and sustaining of attention, planning, organizing and executing tasks. We adopted the definition proposed by Blair (2006), which goes beyond the classical definition of executive functioning (Diamond, 2013) and overlaps with the more comprehensive construct of fluid cognition. This includes speed of processing and working memory, as these cognitive processes co-operate purposefully to organize and execute sequential steps or actions (Alvarez and Emory, 2006). These may be particularly associated with age-related cognitive decline (e.g., Johnson et al., 2007) as well as age-related compensatory mechanisms (Buckner, 2004; Ouwehand et al., 2007). To incorporate these cognitive components in our definition and avoid confusion with terminology, we will use the term “fluid cognition” over “executive function.” Although the unitary entity of fluid cognition is conceptually useful, it is important to notice that different components are distinguishable (for a meta-analytic review see Alvarez and Emory, 2006), and dissociate both in healthy aging and pathological conditions (e.g., Robbins et al., 1998; Piguet et al., 2002; Godefroy, 2003; Brandt et al., 2009; Turner and Spreng, 2012; White, 2013; De Felice et al., 2018). The focus of the present study is to investigate how the behavioral relationships across these different cognitive components vary with age (dispersion index), and whether this reveals specific association with performance in different groups of healthy older adults.

In contrast to individual cognitive performance, the dispersion index is thought to provide an indicator of fairly stable endogenous factors, such as central nervous system (CNS) integrity (MacDonald et al., 2006, 2009), and to be less influenced by situation-dependent factors including fluctuations in stress or sleep (Hultsch et al., 2000). Several studies have reported that a measure of dispersion can be a meaningful indicator of individual differences in behavioral cognitive integrity and neurological mechanisms over age, education and socio-economic status (Hultsch et al., 2000; Rapp et al., 2005; Hilborn et al., 2009; Wojtowicz et al., 2012; Halliday et al., 2018). Behavioral studies have shown that greater dispersion across neuropsychological measures is associated with greater cognitive decline and poorer performance in healthy older adults (Rapp et al., 2005; Hilborn et al., 2009; Wojtowicz et al., 2012; Halliday et al., 2018). However, little attention has been given to the cognitive behavioral profile of older adults who do not show such dispersion, and fewer studies have specifically focused on variability in dispersion between old-age groups (e.g., see Hilborn et al., 2009).

The variance in the degree of dispersion associated with older adults is consistent with models of successful aging such as the Selection, Optimization, and Compensation (SOC) model (Baltes and Baltes, 1990). The SOC model defines successful aging as a heterogenous process of losses and gains, whereby older adults select tasks that are meaningful to them, optimize the resources available to complete those tasks, and engage in compensatory mechanisms to adapt to age-related losses. Variability across individuals in engaging such strategies depends on resources available (Lang et al., 2002; Amieva et al., 2014) and may result in different degrees of “successful” aging. This definition has the advantage of allowing for non-normative, individual cognitive profiles, not necessarily defined by chronological age (e.g., Lang et al., 2002). In fact, the model refers to compensatory strategies including behavioral adjustments to cope with a large range of age-related losses (biological, cognitive, psychological, sensorimotor, socio-economic, etc.). With regards to cognitive losses, compensatory mechanisms may take place unconsciously and manifest behaviorally only when performance is specifically tested (e.g., Li et al., 2001; Silagi et al., 2015).

We argue that mechanisms of cognitive compensation preceding and/or counteracting cognitive decline may have been overlooked in behavioral studies, in favor of an analysis of the variability in individual performance across cognitive domains (dispersion). This may have left unexplored stages in aging when cognitive changes produce distinct patterns of behavioral outcomes, in cases where dispersion is reduced or absent. Dispersion may occur later in aging, is usually associated with decrement in performance and tends to be more easily detectable by neuropsychological tasks. Additionally, behavioral indices of cognitive compensation may tend to remain hidden when older groups are compared to young adults, and when no direct comparisons are carried out within older groups. Although studies on cognitive aging have examined age-related cognitive decline and compensatory mechanisms (Dixon and Bäckman, 1995; Glisky et al., 2001; Dixon and de Frias, 2007; Raz, 2009), and have tried to identify specific factors contributing to cognitive compensation (e.g., working-memory, Borella et al., 2010; Karbach and Verhaeghen, 2018), little attention has been devoted to the study of dispersion and its relation to (good) performance, in the context of ‘successful aging.’ In order to investigate these issues, we specifically compared two older-age groups (middle-old adults in their 60 s and old-old adults over 75 years old) to young controls on a series of tasks engaging fluid cognition.

In line with a progressive neurodegeneration with aging (Raz et al., 2005), we expect middle-old adults to show overall better performance than old-old adults. However, when looking at the dispersion index and performance, we predict that middle-old adults would show a distinct pattern of results compared to both young and old-old adults. Specifically, middle-old adults may show similar dispersion to young adults and less than old-old adults, while showing performance levels in the middle between young and old-old groups. This could possibly reflect greater cognitive stability, resulting in limited dispersion in this age group and better performance. In contrast, the old-old group may show greater dispersion and poor performance compared to the other age-groups, possibly reflecting advanced aging (Li et al., 2004; Sosnoff and Newell, 2006), and/or a stage when compensatory mechanisms may become increasingly difficult to implement as a result of age-related losses in resources (Freund and Baltes, 2002; for a review see Ouwehand et al., 2007).

If the dispersion index within older-age groups is modulated by the degree of engagement in cognitive compensatory strategies (to counteract age-related loss), which in turn depends on resources available (Lang et al., 2002; Amieva et al., 2014), it follows that the older adults with lower dispersion would also be relying on cognitive reserve to a greater extent. Cognitive reserve has been defined as a set of variables including education, intelligence and novelty (Stern, 2002, 2009, 2012), which helps the brain to flexibly adapt to and compensate for pathological loss (Robertson, 2013). It would follow that performance should be positively correlated with factors such as education, specifically in those age-groups that engage in compensatory strategies, but not in those age-groups that either do not need to compensate (e.g., young adults) or whose neurological aging processes have reached an advanced stage where cognitive resources are more limited (Glisky et al., 2001; Sosnoff and Newell, 2006; Ouwehand et al., 2007; Raz, 2009). According to this argument, old-old adults would show the greatest dispersion and this would be coupled with poor-performance, while factors contributing to cognitive reserve (e.g., education level) would have little influence on general performance, in accordance with studies showing that factors considered to be protective against mental aging may no longer benefit the oldest elders (Paulo et al., 2011).

The counter argument would be that middle-old age is not qualitatively different from either young adulthood or old-old age, and differences across groups should simply reflect a linear increase in dispersion and decrease in performance. According to this second hypothesis, the middle-old group should either resemble young adults or old-old adults, depending on where in the developmental trajectory they collocate, thus showing either little dispersion and good performance or significant dispersion and poor performance, respectively. Taking into account evidence that variability between individuals exists, particularly in older groups (Morse, 1993), it may indeed be possible that our middle-old age groups would include both young-like participants as well as old-old-like participants: this would predict negative correlation between dispersion and performance, consistent with a linear increase in dispersion and decrease in performance with aging (e.g., Hilborn et al., 2009). This hypothesis would also predict that an index of cognitive reserve such as educational level should not have any specific effect on any age-group in particular more than another, benefitting either all or none.

Furthermore, if cognitive outcome in aging reflects both cognitive resources available and ability to recruit those resources, it follows that performance should be modulated by task demand. To test this hypothesis, we designed a working memory task based on the findings from an earlier study (Cappell et al., 2010). By using functional magnetic resonance imaging (fMRI), Cappell and Gmeindl found that different patterns of neural recruitment were associated with different levels of performance in healthy older-adults, and reported variations as a function of working-memory load: while lower-loads were associated with greater neural recruitment in older-adults and age-equivalent performance, at higher-loads conditions showed lower recruitment as well as poor performance. Although this study was intended to investigate age-related differences in neural recruitment, and we are instead interested in age-related variance in cognition, we still believe that the logic of comparing performance for different working-memory loads is relevant to test our hypothesis. In this task, working-memory span does not increase based on individual performance (as in classic digit span tasks), but participants are given different fixed span length (working-memory loads) and performance is compared across conditions. If the middle-old group presents a distinct cognitive profile, with behavioral performance reflecting age-related changes in cognitive recruitment, we would expect different patterns of results for different working-memory loads: for lower loads, the middle-old group would show young-equivalent performance, while performing better than old-old adults. However, higher loads would be so cognitively demanding that middle-old and old-old adults would perform almost equally poorly. In contrast, if middle-old adults are at an intermediate stage between young and old-old adults, one should expect the middle-old group to perform worse than young adults and better than old-old adults across conditions, although differences in age-related change in each of the measures may result in some variance. See **Table 1** for a schematic summary of these hypotheses.

**Table 1:** Summary of the hypotheses.

Hypothesis	Dispersion index	Performance	Effect of cognitive reserve factors (e.g., education) on performance
A. Cognitive aging reflects qualitatively different developmental stages	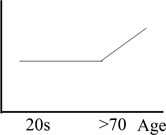	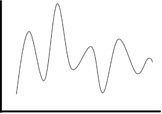	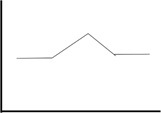
B. Cognitive aging reflects a linear decline in performance	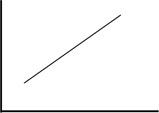	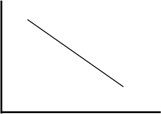	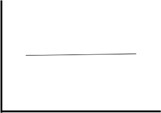

*The two hypotheses are presented with reference to their specific predictions of age-related changes in dispersion index, performance and effect of indices of cognitive reserve such as educational level. (A) Cognitive aging reflects qualitatively different developmental stages: this hypothesis interprets the dispersion index to reflect age-related differences in neural recruitment and predicts that this would increase with aging in a non-linear manner, depending on the efficiency of compensatory mechanisms/severity of neural noise; it would follow that performance may vary depending on task demand (compensatory strategies may overcome aging losses only until a certain threshold) and that indices of cognitive reserve may specifically improve performance when compensatory strategies are employed but not when these are neither necessary (young age) nor feasible (advanced aging). (B) Cognitive aging reflects a linear decline in performance: this hypothesis predicts a linear increase in dispersion with aging, as this is reflecting increase in neural noise, which in turn would results in linear decrease in performance, with factors contributing to cognitive reserve failing to show beneficial effect which are specific to any age group or cognitive styles.*

## Materials and Methods

### Participants

Twenty-one young (22–31 years old), 20 middle-old (59–71 years old), and 20 old-old (76–91 years old) participants volunteered in the present study (for sample demographic information see **Table 2**). Groups were matched as far as possible on gender and educational level, with no significant difference between age groups on education variable (*p* = 0.68). All participants were native Italian speakers.

**Table 2:** Demographic data.

	Sample size (n)	Gender M: F	Age (years)			Education (years)		
			Mean	SD	Range	Mean	SD	Range
Young	21	12:9	25.14	±2.85	22–31	14.33	±2.67	9–18
Middle-Old	21	11:10	65.05	±4.19	59–73	14.05	±4.04	8–19
Old-Old	20	11:9	80.65	±4.38	76–91	13.40	±3.57	5–18

*M: male; F: female.*

Participants were excluded only on the basis of factors which could significantly affect their ability to do the tasks: e.g., history of stroke; neurological impairments; serious cognitive, visual, hearing or motor impairment. The cognitive status of older adults was determined based on self-report. Older participants included in the final sample were community dwelling adults presumed cognitively normal, they were all non-institutionalized and independent-living individuals. None had a diagnosis of Mild Cognitive Impairment or Dementia. None had any difficulty with performing the tasks.

### Tasks

The aim is to investigate variability in performance across measures of fluid cognition (see Blair, 2006). Within this definition, we selected neuropsychological tests that are considered measures of psychomotor speed [e.g., Trial Making Test part A (TMT-A), Arbuthnott and Frank, 2000; Salthouse, 2011], short-term memory and executive-attention (e.g., Digit Span Forward, Kane and Engle, 2002) and more classic executive function tasks engaging working-memory (e.g., Digit Span Backward, Kane and Engle, 2002), switching and inhibition (TMT part B/A, Salthouse, 2011; verbal fluency, Troyer et al., 1998; Shao et al., 2014). The common denominator here is cognitive control (Morton et al., 2011): we selected tasks which are considered to engage a series of cognitive processes that co-operate purposefully, by organizing and executing sequential steps or actions (Alvarez and Emory, 2006; Blair, 2006), and that may be particularly associated with age-related cognitive decline (e.g., Johnson et al., 2007) as well as age-related compensatory mechanisms (Buckner, 2004; Ouwehand et al., 2007).

#### Digit Span (DS): Forward and Backward

Participants are required to recall an increasingly long sequence of digits in the same order (DS forward) or in reverse order (DS backward) (Wechsler, 2008). The two versions of this task provide measures of different components of working memory (Baddeley, 1983), including maintenance of information online (DS forward) and manipulation of such information (DS backward). Although other models consider DS forward as a measure of verbal short-term memory separated from working-memory (see Warrington and Shallice, 1969), what is important here is its conceptualization within fluid cognition (Schofield and Ashman, 1986) and executive-attention (Kane and Engle, 2002).

#### Trail Making Test (TMT)

This test is widely used as a measure of both speed of processing (Part A) and executive functions (Part B) (Reitan and Wolfson, 2009). This paper and pencil task requires completion of sequences as fast as possible by joining different circles. In part A (TMT-A), participants are asked to link numbers 1–25 in ascending order; in part B (TMT-B), participants are asked to link numbers and letters in alternating order (e.g., 1-A; 2-B; 3-C…). Time (sec) to complete TMT-A is taken as a measure of psychomotor speed, while the TMT-B over TMT-A ratio (B/A) is computed as an index of executive processing. This has been shown to be a better measure of executive function compared to the TMT-B minus TMT-A difference (Salthouse, 2011), and to specifically reflect switching and inhibition (Arbuthnott and Frank, 2000). Each participant was given one practice trial before each component.

#### Verbal Fluency (VF): Phonemic and Semantic

This test provides measures of executive processing including lexical retrieval, inhibition, mental set-shifting, internal response generation, updating and self-monitoring (Benton and Hamsher, 1978; Shao et al., 2014). In the phonemic task, participants are given a letter (P, L, or F) and asked to produce as many words starting with that letter as they can in a minute. Participants are told that they cannot produce proper names (e.g., cities, people, etc.), and that they will not receive a score for alterations of the same word [e.g., casa (house) and *casetta (little house)*]. In the semantic task, participants are given the semantic category “animals” and are asked to produce as many words within that category as they can in a minute. These two versions of verbal fluency have been shown to reflect clustering (generating words within subcategories) and switching (alternating between subcategories) in slightly different degrees, with dissociations being reported in clinical populations (Juhasz et al., 2012) and different neural correlates being associated with these two conditions (Troyer et al., 1998).

#### Word Span

This task was designed based on a previous study (Cappell et al., 2010) specifically to have a measure of working memory as a function of different working-memory loads. To control for any possible practice effect with the digit span task, as well as to account for differences between digit and word span tasks found in early studies (Brener, 1940; Crannell and Parrish, 1957), we changed the span stimuli from digits to words. This is believed to improve sensitivity of the measure (e.g., Kochhann et al., 2009), which may be particularly important in the case of fixed working-memory span (rather than adjusting online task difficulty based on individual performance). The researcher reads aloud three lists of words, one with four words, another with five words, and another with seven words, once for each condition. Word stimuli were selected from the Burani et al. (2011) database for Italian words and matched for: frequency; mean bigram-frequency; letter length; syllable length; and mean reading-time. Participants are asked to repeat the list in the same serial order. A score of 2 is given for each item recalled in the correct ordinal position, 1 for each item recalled in an incorrect ordinal position and 0 for items not recalled (this gives a maximum score of 8 for word span 4, 10 for word span 5, and 14 for word span 7).

### Intra-Individual Variability Measure: Dispersion Index

To investigate whether intra-individual variability across tasks can represent a meaningful index of cognitive styles in healthy aging, a dispersion index was calculated for each individual and then used in further analysis (see below). This measure reflects performance variability across cognitive measures within an individual.

There are multiple indices that can be computed as a measure of intra-individual variability (Slifkin and Newell, 1998). The simplest of these is the intra-individual standard deviation (iSD), which is calculated as the standard deviation across standardized scores of different tasks for a single individual. This measure can be problematic when there are significant systematic group differences in average level of performance, as greater means tend to be associated with bigger variance (Hale et al., 1988; Stawski et al., 2017). This can represent a serious confound in the case of comparison of performance between age-groups. To control for differences in variability that may derive from individual mean-level performance, the coefficient of variation (COV) can be calculated by dividing the iSD by the mean of performance for each individual. However, it has been shown that this measure is less sensitive to the pure endogenous factors defining individual cognitive structure, as it does not control for systematic confounds across individuals and/or groups, such as gender or boredom (Wojtowicz et al., 2012). Instead, the dispersion index, although requiring a slightly longer computation, is thought to provide a more reliable measure of CNS integrity and index of individual cognitive structure (Hultsch et al., 2002; Wojtowicz et al., 2012).

Individual dispersion profiles are obtained by using a regression technique, which computes iSD scores from standardized test scores (Christensen et al., 1999; Hultsch et al., 2002; Cole et al., 2011; Halliday et al., 2018). Test scores of interest (TMT-A, TMT-B/TMT-A, Digit Span Forward, Digit Span Backward, Verbal Fluency Phonemic, Verbal Fluency Semantic, Word Span average across trials 4, 5, and 7) were initially regressed on linear age trends across all participants, then the resulting residuals from these models were standardized as z-scores (*M* = 0, *SD* = 1), with individual iSDs subsequently computed across these *z*-scores. Higher values in dispersion index reflect greater intra-individual variability in cognitive functions, across tasks.

### Procedure

Participants were recruited through advertising flyers in the local community. Each session took approximately 30 min. The protocol was approved by the Aston University ethics committee. All participants gave written informed consent in accordance with the Declaration of Helsinki. All written information was in a font clear and big enough for all age-groups. To facilitate comprehension, instructions were presented both in writing and orally. Tasks were presented in the following order: Trail Making Test (TMT), Digit Span (DS; Forward and Backward), Verbal Fluency (VF; Phonemic and Semantic), and Word Spans (WS). The same order was followed with all participants. This order was designed to have a balanced cognitive demand, without over-stressing the same ability consecutively (e.g., working memory in Digit Span and Word Span).

### Statistical Analysis

#### Performance Across Groups: Is the Middle-Old Age Simply Reflecting an In-Between Stage in Cognitive Aging?

To examine the age-related differences in different cognitive measures, a series of one-way between-subject ANOVA will be conducted to compare performance in each task between age groups. When an overall significant effect of age is found, separate independent *t*-tests will be computed to examine differences between groups. No correction for multiple comparisons will be made, because the aim is to identify cognitive measures for which there is a difference between groups, rather than testing the null hypothesis of no overall difference between the groups (Armstrong, 2014). Also, the conservative nature of *post hoc* tests would increase the risk of Type II error (Wuensch, 2017). Moreover, a 3 (age-group) × 3 (word span loads) ANOVA will be conducted to examine whether the differences in performance between the different levels of load in the word span task were different for the different age groups. This will be done using percentages because of the different range of possible scores for each load.

#### Comparison of the Dispersion Index Across Groups and Relation With Performance and Educational Level

First, independent student *t*-tests will be computed to examine age-group differences in dispersion index. Second, to test the hypothesis that the link between performance and dispersion presents some qualitative differences between the middle-old and the old-old group and to examine the role of reserve, we will perform a series of correlation analysis between performance and (i) dispersion index (iSD) and (ii) education (an index of cognitive reserve, see Introduction) across age-groups separately as well as for the whole sample.

## Results

### Performance Across Groups: Is the Middle-Old Age Simply Reflecting an In-Between Stage in Cognitive Aging?

**FIGURE 1:**
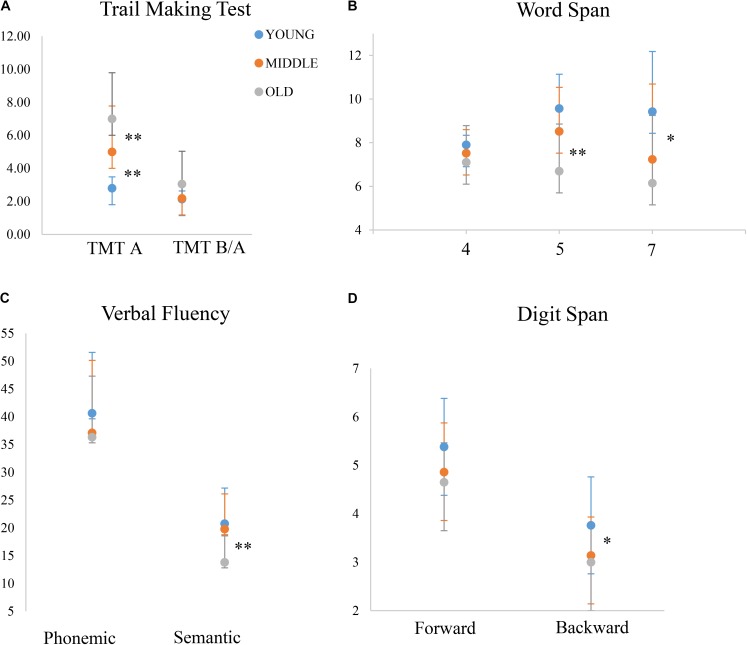
Performance of the three age-groups on different tasks. *y*-axis depicts the outcome of each given test, either in terms of time **(A)** or score **(B–D)**. The middle-old group performs similarly to the young-group on certain tasks, while similarly to the old-old group on others. Note that for TMT B/A, the young and the middle-old group’s performances overlap almost completely. ^∗^*p* ≤ 0.05; ^∗∗^*p* ≤ 0.01; for visualization purposes, this figure only shows significant differences between the middle-old group and the other two groups, while differences between the young and the old-old group are omitted.

Results are summarized in **Table 3** and **Figure 1**.

**Table 3:** One-way ANOVA between the Middle-Old group and the Young and the Old-Old groups on different tasks.

Group mean (SD)	Young	vs.	Middle-Old	vs.	Old-Old	vs. Young
DS forward (score)	5.38 (1.12)		4.86 (1.01)		4.65 (0.81)	^∗^
DS backward (score)	3.76 (1.09)	^∗^	3.14 (0.79)		3 (0.73)	^∗^
TMT A (sec)	27.67 (6.83)	^∗∗^	49.57 (27.73)	^∗∗^	70.20 (27.86)	^∗∗∗^
TMT B/A (sec)	2.14 (0.49)		2.18 (0.74)		3.05 (1.98)	^∗^
VF phonemic (score)	40.62 (10.95)		37.10 (13.03)		36.30 (11)	
VF semantic (score)	20.76 (6.39)		19.10 (6.35)	^∗∗^	13.80 (4.75)	^∗∗∗^
WS 4 (score)	7.90 (0.64)		7.52 (1.08)		7.10 (1.68)	
WS 5 (score)	9.57 (1.57)		8.52 (2.02)	^∗∗^	6.70 (2.15)	^∗∗∗^
WS 7 (score)	9.43 (2.75)	^∗^	7.24 (3.45)		6.15 (3.10)	^∗∗^

*Significant differences obtained between the Middle-Old group and the other two groups are reported under the ‘vs.’ columns. t-test ^∗^p ≤ 0.05; ^∗∗^p ≤ 0.01; ^∗∗∗^p ≤ 0.001. TMT, trail making test; DS, digit span; VF, verbal fluency; WS, word span.*

#### Digit Span

There was a significant group effect on performance on the Digit Span Forward component [*F*(2, 59) = 2.97, *p* = 0.05], with a significant difference between the young and the old-old group [young *M* = 5.38; old-old *M* = 4.65; *t*_(39,41)_ = 2.38, *p* = 0.02, 95% CI (0.11 and 10.35)]. The middle-old group (*M* = 4.86) did not significantly differ from the other two groups (vs. young *p* = 0.12; vs. old-old *p* = 0.48).

There was also a significant group effect on performance on the Digit Span Backward component [*F*(2, 59) = 4.32, *p* = 0.01]. The young group significantly differed from the old-old group [young *M* = 3.76; old-old *M* = 3; *t*_(39,41)_ = 2.61, *p* = 0.01, 95% CI (0.17 and 1.35)]. The middle-old group significantly differed from the young group [*M* = 3.14; *t*_(39,41)_ = 2.10, *p* = 0.04, 95% CI (0.02 and 2.21)], but not from the old-old group (*p* = 0.55).

#### Trail Making Test (TMT)

There was a significant group effect on performance on the TMT-A [*F*(2, 59) = 17.62, *p* = 0.0001]. The mean scores for the three age-groups (young *M* = 27.67; middle-old *M* = 49.57; old-old *M* = 70.20) significantly differed from each other [young vs. middle-old *t*_(39,41)_ = −3.51, *p* = 0.001, 95% CI (−34.49 and −9.31); young vs. old-old *t*_(39,41)_ = −6.78, *p* = 0.0001, 95% CI (−55.20 and −29.86); middle-old vs. old-old *t*_(39,41)_ = −2.37, *p* = 0.02, 95% CI (−38.19 and −3.06)].

There was a significant group effect on performance on the TMT-B/TMT-A ratio [*F*(2, 59) = 3.45, *p* = 0.03]. We found a significant difference between the young and the old-old group [young *M* = 2.13; old-old *M* = 3.05; *t*_(39,41)_ = −2.03, *p* = 0.05, 95% CI (−1.8 and −0.003)]. The middle-old group (*M* = 2.18) did not significantly differ from the other two groups (vs. young *p* = 0.82; vs. old-old *p* = 0.07).

#### Verbal Fluency

There was no significant group effect on performance on the Phonemic Verbal Fluency task [*F*(2, 59) = 0.79, *p* = 0.45].

There was a significant group effect on performance on the Semantic Verbal Fluency task [*F*(2, 59) = 7.73, *p* = 0.001]. The young group (*M* = 20.76) differed from the old-old group [*M* = 13.80; *t*_(39,41)_ = 3.94, *p* = 0.0001, 95% CI (3.38 and 10.53)]. The middle-old group (*M* = 19.10) differed from the old-old group [*t*_(39,41)_ = 3.01, *p* = 0.005, 95% CI (1.73 and 8.85)], but not from the young group (*p* = 0.40).

#### Word Span

This task was designed to test whether differences in performance across groups remained stable across conditions or instead varied as a function of working-memory load. We found ceiling effects for Word Span 4 (max score = 8, young *M* = 7.90 and 95% scoring the maximum; middle-old *M* = 7.52 and 80% scoring the maximum; old-old *M* = 7.10 and 75% scoring the maximum). Therefore, we did not include this condition in the further analysis. There was also a ceiling effect for the young group for Word Span 5 (90% scored the maximum of 10) and so this group was omitted from the within subjects ANOVA. The 2 (age-group) × 2 (word span loads 5 and 7) ANOVA found an overall effect of level of demand [*F*(1,39) = 51.37, *p* < 0.001] and an effect of age group: *F*(1,39) = 5.21, *p* < 0.05, but there was no statistically significant interaction between the effects of age and working-memory load condition on performance, *F*(1,39) = 1.76, *p* = 0.193.

We then wanted to test whether there were any group differences for each working memory load separately. There was no significant group effect on performance on the Word Span 4 [*F*(2, 59) = 2.42, *p* = 0.09]. This may be due to ceiling effects.

There was a significant group effect on performance on the Word Span 5 [*F*(2, 59) = 11.63, *p* = 0.0001]. The young group (*M* = 9.57) significantly differed from the old-old group [*M* = 6.67; *t*_(39,41)_ = 4.89, *p* = 0.0001, 95% CI (1.68 and 4.05)]. The middle-old group (*M* = 8.52) significantly differed from the old-old group [*t*_(39,41)_ = 2.80, *p* = 0.008, 95% CI (0.50 and 3.14)], but not from the young group (*p* = 0.07), although bearing in mind possible ceiling effects for the young group (max score = 10; percentages for participants scoring the maximum: young 90%, middle-old 57%, old-old 20%).

There was a significant group effect on performance on the Word Span 7 [*F*(2, 59) = 5.93, *p* = 0.004]. The young-group (*M* = 9.43, *SD* = 2.75) significantly differed from the old-old group [*M* = 6.15, *SD* = 3.10; *t*_(39,41)_ = 3.58, *p* = 0.001, 95% CI (1.43 and 5.12)]. The middle-old group (*M* = 7.24, *SD* = 3.45) showed a significant difference from the young group [*t*_(39,41)_ = 2.27, *p* = 0.02, 95% CI (−0.24 and −4.13)], but not from the old-old group (*p* = 0.30). These results do not seem to be due to ceiling effects (max score = 14; percentages for participants scoring the maximum: young 0.09%, middle-old 0.09%, old-old 0%).

Taken together, these results indicate that the middle-old group showed a mixed pattern of performance, resembling the young-group on certain tasks and the old-old group on others (see **Figure 1**).

### Intra-Individual Variability Across Tasks: The Dispersion Index

**FIGURE 2:**
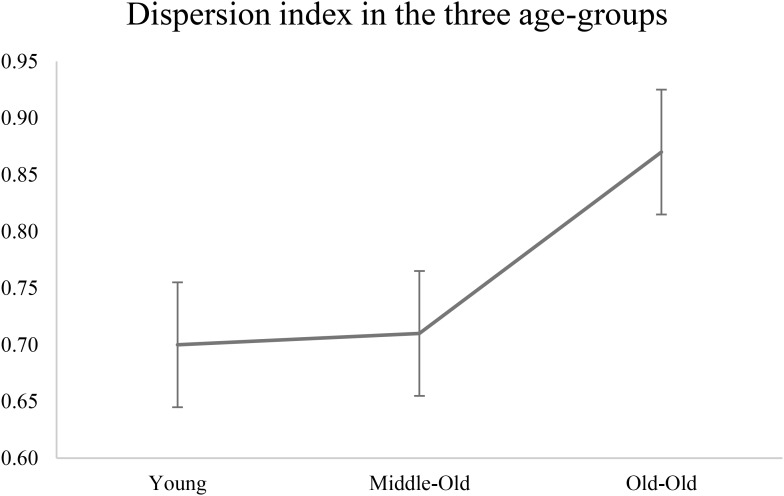
Dispersion index plotted for the three age-groups. The graph shows a non-linear increase in dispersion index as a function of age groups (see **Table 1**, hypothesis A). Young adults aged 22–31; Middle-Old adults aged 59–71; Old-Old adults aged 76–91 (independent *t*-test young vs. middle *p* = 0.93; middle vs. old-old *p* = 0.08; young vs. old-old *p* = 0.10).

Average dispersion index was calculated for each age group separately: the young group had a dispersion index score of 70 (*SD* = 0.30), the middle-old group of 71 (0.23), and the old-old group of 87 (0.35). Although not reaching significance (independent *t*-test young vs. middle *p* = 0.93; middle vs. old-old *p* = 0.08; young vs. old-old *p* = 0.10), this pattern of results reveals a bigger dispersion index associated with the oldest group. **Figure 2** shows dispersion index as plotted for the three age groups, which follows a non-linear increase with age.

We questioned whether impairment may have been associated with a specific sub-group of tests, so that a specific pattern of performance may have introduced a confound in the way variability across tasks has been computed. **Table 4** shows individual standardized scores across tests for the whole sample. Performance does not systematically drop for some measures: rather, impairment is more generally seen across the neuropsychological test battery. A factorial ANOVA was also carried out to test for the main effect of cognitive test on score. Results show no significant main effect of cognitive test(*F*(8, 531) = 0.01, *p* = 1), nor significant age^∗^test interaction effect (*F*(2, 531) = 0.42, *p* = 0.97).

**Table 4:** Standard scores across tasks for the whole sample.

Group	WS4	WS5	WS7	TMT A (reversed)	TMT B/A (reversed)	DS forward	DS backward	VF phonology	VF semantic
Y	0.08	0.21	−0.78	0.02	0.02	−0.35	−0.85	−0.02	−0.04
Y	0.07	0.19	–1.12	−0.15	0.73	0.65	0.28	0.49	1.60
Y	0.07	0.19	0.83	0.16	−0.43	−0.37	−0.87	0.32	−0.06
Y	0.08	0.21	0.85	0.23	−0.44	0.66	1.44	0.32	−0.21
Y	0.13	0.29	−0.38	−0.30	0.32	–1.32	−0.79	–1.04	−0.30
Y	0.13	0.29	0.27	0.01	0.09	0.71	−0.79	0.26	–1.30
Y	0.09	0.23	0.86	0.18	−0.35	–1.36	−0.84	0.76	0.81
Y	0.10	0.25	1.53	−0.01	0.38	3.74	3.76	1.81	2.65
Y	0.09	0.23	–1.09	0.13	−0.14	–1.36	−0.84	0.50	−0.19
Y	0.05	–0.85	−1.16	−0.43	0.23	0.63	0.25	−0.39	−0.92
Y	0.14	0.31	−0.69	−0.14	0.05	−0.29	−0.77	1.05	−0.78
Y	0.05	0.15	1.44	–1.08	0.22	−0.39	1.39	–2.12	–2.25
Y	0.05	0.15	0.79	0.23	−0.98	0.63	0.25	−0.47	−0.26
Y	0.06	0.17	0.16	−0.27	−0.79	0.64	0.26	–1.76	−0.91
Y	–1.67	0.15	0.79	−0.21	0.11	–1.41	1.39	−0.30	0.24
Y	0.15	0.33	–1.32	0.38	−0.07	0.74	0.39	0.45	0.89
Y	0.05	0.15	0.14	0.14	−0.28	0.63	−0.90	0.13	−0.43
Y	0.08	0.21	0.20	0.15	0.16	−0.35	0.29	0.67	0.12
Y	0.08	0.21	−0.13	−0.25	−0.25	−0.35	–2.00	–1.15	–1.37
Y	0.10	0.25	−0.74	0.08	0.17	−0.33	−0.82	−0.01	0.66
Y	0.05	–3.34	−0.18	0.01	0.06	−0.39	0.25	1.17	0.40
MO	0.57	1.14	–1.24	0.79	0.57	0.21	1.02	−0.17	−0.74
MO	0.44	0.90	0.49	0.72	0.71	1.08	0.83	1.40	2.21
MO	0.50	1.00	0.26	0.31	0.24	−0.89	−0.23	−0.65	−0.69
MO	0.48	−0.51	−0.41	0.85	0.47	0.11	0.90	−0.66	−0.05
MO	–2.97	0.96	−0.43	0.64	−0.75	−0.92	–1.41	−0.84	−0.23
MO	0.52	1.04	1.59	0.98	0.69	1.17	–1.35	1.27	1.00
MO	0.52	1.04	2.24	1.16	0.06	2.18	0.94	2.91	2.83
MO	–1.13	–2.30	–2.18	–3.51	–1.01	–1.80	−0.09	–1.37	−0.87
MO	0.55	1.10	0.35	0.68	0.28	−0.83	−0.15	0.51	0.72
MO	0.45	−0.57	−0.47	0.58	0.76	−0.94	−0.30	0.10	0.07
MO	0.46	–1.05	−0.77	0.17	0.75	−0.93	−0.28	0.28	−0.91
MO	0.46	0.94	−0.77	1.22	0.63	1.11	0.86	0.02	1.75
MO	–1.23	−0.99	–1.69	–1.00	−0.61	−0.89	–1.38	–1.34	−0.53
MO	0.48	0.98	0.89	0.15	1.15	0.11	0.90	1.68	1.61
MO	0.47	–1.03	–1.08	–2.50	0.37	−0.92	–1.41	0.38	0.27
MO	0.54	0.09	0.98	0.78	0.12	0.17	−0.17	−0.11	0.37
MO	0.57	1.14	0.38	0.53	−0.44	1.23	−0.12	−0.17	0.09
MO	0.45	−0.07	0.18	0.27	0.97	0.08	0.85	–1.45	−0.26
MO	–1.17	1.10	0.67	0.55	0.58	0.19	−0.15	−0.19	0.72
MO	0.56	0.13	1.67	0.76	0.37	−0.82	1.01	−0.96	−0.09
MO	0.50	1.00	−0.07	0.70	0.17	1.14	–1.38	–1.26	−0.20
OO	0.71	1.41	–1.01	0.45	−0.34	−0.65	0.08	0.42	–1.67
OO	0.70	0.39	−0.37	−0.89	−0.40	0.36	1.21	1.02	–1.19
OO	0.71	0.41	1.27	0.06	1.29	1.39	0.08	−0.62	−0.35
OO	0.63	−0.72	−0.16	1.19	0.13	1.30	1.12	1.07	−0.97
OO	0.63	–1.72	0.17	–1.42	0.20	0.28	−0.03	–1.00	0.53
OO	0.63	0.27	–1.13	–1.42	1.24	−0.73	−0.03	0.64	–1.30
OO	0.66	−0.66	−0.43	0.20	−0.23	0.32	0.02	–1.42	−0.42
OO	0.76	1.51	0.39	1.04	0.04	1.45	1.31	0.63	−0.09
OO	–1.07	–1.68	0.20	0.16	0.00	−0.71	0.00	0.22	−0.27
OO	0.64	–1.20	0.51	1.40	0.12	−0.72	−1.16	−0.57	0.21
OO	−1.96	−0.74	−1.48	−3.73	−0.69	−1.76	−1.19	−1.01	−1.15
OO	−2.73	−0.56	−1.31	1.35	−0.34	0.38	0.10	1.38	0.83
OO	−3.67	−0.72	−2.11	−0.77	−0.30	−0.73	−0.03	−0.74	−0.80
OO	0.66	1.33	−0.10	0.37	−0.02	0.32	0.02	−0.38	0.08
OO	0.78	1.55	1.40	−0.42	1.48	0.46	0.19	0.55	1.27
OO	−2.81	−1.22	0.49	0.54	−2.00	−0.73	1.12	0.73	0.53
OO	0.72	0.44	1.94	−1.35	0.61	0.38	−1.05	−0.78	0.34
OO	0.65	−1.68	−0.12	0.64	0.01	−0.71	−1.14	1.60	0.40
OO	0.64	−0.70	1.16	−1.13	0.50	0.30	1.13	−0.13	−0.12
OO	0.63	−1.72	−0.16	0.02	−6.20	−0.73	−1.17	−1.70	−1.30

*The table shows standardized scores for all participants in different age groups for each neuropsychological test. There is no specific pattern of one test or sub-group of tests being specifically represented with low scores in any age group. Rather there is a general variability across the neuropsychological battery. Red scores are < − 1. Y, young group; MO, middle-old group; OO, old-old group; WS, word span; TMT, trail making test; DS, digit span; VF, verbal fluency.*

### Comparison of the Dispersion Index Across Groups and Relation With Performance and Educational Level

Some significant correlations emerged when considering dispersion index and performance on different tasks and educational level across groups. Results are summarized in **Table 5** and significant correlations are also reported below for each group.

**Table 5:** Correlations between dispersion index and education and performance in different tasks.

	Young		Middle-Old		Old-Old	
	iSD	Education	iSD	Education	iSD	Education
DS forward (score)	0.403	−0.045	−0.020	0.429	−0.279	0.196
DS backward (score)	**0.657**^∗∗^	0.132	−0.143	0.325	−0.343	0.041
TMT A^†^ (sec)	−0.296	0.169	0.101	**0.545^∗^**	0.061	**0.598^∗∗^**
TMT B/A^†^ (sec)	0.211	0.232	−0.172	0.401	**−0.755^∗∗∗^**	0.129
VF phonemic (score)	0.083	0.394	0.083	**0.652^∗∗^**	−0.161	0.310
VF semantic (score)	0.092	0.248	0.010	0.409	−0.208	0.110
WS 4 (score)	−0.098	0.114	0.104	0.418	−0.101	0.098
WS 5 (score)	−0.078	−0.084	−0.298	**0.463^∗^**	−0.350	0.153
WS 7 (score)	**0.580^∗∗^**	0.211	−0.002	**0.480^∗^**	−0.063	0.132
Overall performance	0.403	0.278	−0.120	**0.661^∗∗^**	**−0.499^∗^**	0.373

*Significant results are in bold. ^∗^p ≤ 0.05; ^∗∗^p ≤ 0.01, ^∗∗∗^p ≤ 0.001. ^†^Sign has been sign-reversed here for consistency with the other tests, so that all correlations are presented in the same direction. iSD (intra-individual SD, an index of dispersion across all the tasks: the higher the iSD, the higher the dispersion); DS, digit span; TMT, trail making test; VF, verbal fluency; WS, word span.*

#### The Young Group

Significant positive correlations were found only between iSD and two cognitive measures: score on the Digit Span Backward (*r* = 0.66, *p* = 0.001) and score on the Word Span 7 (*r* = 0.58, *p* = 0.006). In other words, young adults who showed higher dispersion, also showed better performance in these two tasks. Educational level did not correlate with any cognitive measures.

#### The Middle-Old Group

iSD did not correlate with any cognitive measures. Educational level negatively correlated with time on the TMT A (*r* = −0.54, *p* = 0.01) and positively correlated with performances on VF Phonemic (*r* = 0.65, *p* = 0.001), Word Span 5 (*r* = 0.46, *p* = 0.03), Word Span 7 (*r* = 0.48, *p* = 0.02), as well as overall performance, calculated as the average across z-score of the measures of interest (*r* = 0.66, *p* = 0.001). Therefore, while dispersion index was not associated with performance in any of the cognitive measures included in this study, higher levels of education was associated with faster and better performance in this age-group.

#### The Old-Old Group

iSD was positively correlated with time on the TMT A (*r* = 0.75, *p* = 0.0001) and negatively correlated with overall performance, calculated as the average across z-scores of the measures of interest (*r* = −0.50, *p* = 0.02). Therefore, higher dispersion was associated with a decrease in speed of processing and poorer performance in this group. Educational level was negatively correlated with time on TMT A (*r* = −0.60, *p* = 0.005) only, meaning that more years of education were associated with faster completion of the task. No other significant correlations were found between educational level and any of the cognitive measures considered in this study.

## Discussion

The aim of this study was to investigate age-related differences in intra-individual variability, also known as dispersion, across fluid cognition, and to examine the potential of this index to reveal changing patterns of cognitive functioning in later life. We compared performance of young adults in their 20 s, middle-old adults in their 60 s and old-old adults over 75 years old on a number of tasks engaging fluid cognition. Previous studies have shown some inconsistencies in the degree of dispersion found in old age (Hultsch et al., 2000; Li et al., 2004; Rabbitt et al., 2004; Hilborn et al., 2009; Halliday et al., 2018). Dispersion has generally been associated with poor performance and advanced age-related cognitive decline (Rapp et al., 2005; Hilborn et al., 2009). Likewise, and importantly for cognitive theories of compensation (Baltes and Baltes, 1990; Ouwehand et al., 2007), measures of cognitive control have been found to be particularly involved in deployment of resources in the aging brain (Dixon and Bäckman, 1995; Glisky et al., 2001; Stuss et al., 2003; Dixon and de Frias, 2007; Davis et al., 2008; Hilborn et al., 2009; Wojtowicz et al., 2012). We asked whether age-related differences in dispersion across measures of fluid cognition reflect differences in the efficiency of recruitment of resources (cognitive control) in healthy older adults.

When considering between-group differences in dispersion index, our results failed to reach statistically significance, possibly due to small sample size or a limited number of cognitive measures used to compute the dispersion index. However, we observed a general non-linear trend showing that a higher level of dispersion was associated with the oldest group, while the young and the middle-group exhibited very similar dispersion index (see **Figure 2** and results). Moreover, dispersion index in the old-old group was significantly negatively correlated with overall performance, in line with previous studies (Rapp et al., 2005; Hilborn et al., 2009). In contrast, performance in the middle-old group showed a mixed pattern: middle-old adults performed better than the old-old adults and similarly to the young adults on a number of tasks, but also worse than the young adults and similar to the old-old adults on other tasks, possibly depending on differences in cognitive demand across tasks. We argue that this reflects differences in cognitive deployment of resources in aging: specifically, compensatory mechanisms in the middle-old group may have resulted in young-equivalent dispersion across measures of fluid cognition and overall better performance compared to old-old adults.

Although we did not specifically test for compensatory strategies, this interpretation is consistent with the fact that higher educational level – thought to increase cognitive reserve, which in turn supports cognitive compensation (Stern, 2009, 2012) – was related to better performance in the middle-old group only. Moreover, the correlation analysis reveals that the dispersion index may be specifically associated with performance in the old-old group, but not in the other groups. The current pattern of data therefore reveals that the dispersion index can be a useful indicator of cognitive aging, in accordance with previous studies (Hultsch et al., 2002; Halliday et al., 2018), and goes beyond previous work in suggesting that it can be used to study variability in cognition in different cohorts. Importantly, the study of the relationship between dispersion index, performance and cognitive reserve has allowed the emergence of a distinct pattern in which older adults with overall better cognition uniquely benefit from greater cognitive reserve and show young-equivalent dispersion index. We will now discuss specific points which support these conclusions.

### The Middle-Old Group Showed a Distinct Pattern of Performance Compared to the Other Age-Groups

The comparison of three age groups revealed cognitive profiles specific to each age group. This failed to follow a linear decrease in cognitive abilities with increasing age. Although overall results are in line with a progressive neurodegeneration with aging (Raz et al., 2005) – the young and the old-old group being at the two ends of the performance distribution – a closer look at the middle-old group revealed a distinct cognitive profile associated with this cohort. Performance of the middle-old group did not simply fit a stage in-between the good performance of younger adults and the poor performance of older adults, but rather, resembled the performance of each of these two cohorts in different tasks (see **Figure 1**). Specifically, while the old-old group performed significantly worse than younger adults in almost all measures, the middle-old group did not differ from young adults in a series of measures including verbal fluency and short-term memory, and performed better than the older adults in a lexical selection task (verbal fluency semantic) and in a visual search task engaging speed of processing (TMT-A). However, on a working-memory task (Digit Span backward) the middle-old group performed similarly to the old-old group and significantly worse than young adults.

The most distinct pattern of performance in the middle-old group was observed in those tasks requiring a higher level of cognitive control (TMT B/A and verbal fluency), while for tasks that are considered more “automatic,” the cognitive decline across groups was more even (e.g., TMTA, see Miyake et al., 2000 for a discussion on degree of cognitive control within executive functioning). We interpret discrepancy in performance in the middle-old group as reflecting variations in the employment of cognitive resources in different tasks, based on task difficulty and/or resources available to compute the cognitive goal. This interpretation is consistent with previous studies showing that older adults vary in a non-linear manner, either performing good (young-equivalent) or significantly worse than their younger counterparts (e.g., Cabeza et al., 2002; Hultsch et al., 2002).

One could argue that differences across tasks are likely to reflect dissociations in cognitive modules, so that separate executive processes may undergo slightly distinct degeneration progress (Buckner, 2004; Stuss, 2011). However, when taking into account the Word Span task, this interpretation seems unlikely to explain our results: here, cognitive demand was manipulated within the same task. Results revealed some differences across conditions. While little can be said for word span 4, where ceiling effects may have prevented any age-group differences to emerge, in word span 5 the middle-old group performed as well as young adults (although again the performance range of the young adults may have been truncated due to ceiling effects) and significantly better than the old-old adults. However, at higher load (word span 7) they performed significantly worse than the young group and as bad as the old-old group. Our interpretation – although speculative, as we did not test compensatory strategies directly – is that the middle-old group still had enough resources to compensate in some of the tasks (or conditions, in the case of word span), thus exhibiting young-equivalent performance, until a stage at which cognitive demand was too high and performance dropped. In contrast, participants in the old-old group might have reached resource ceiling at an earlier stage, resulting in worse performance than both young and middle-old adults at word span 5 condition.

Although this is not a neural imaging study, these findings are what may be expected behaviorally from the CRUNCH (Compensation-Related Utilization of Neural Circuits Hypothesis, Reuter-Lorenz and Cappell, 2008). This recognizes a trade-off between compensatory potential and task demand. Consistent with the interpretation that variability in performance reflects differences in recruitment of resources and employment of compensatory strategies, using a similar task to the one we designed here, in their fMRI study, Cappell et al. (2010) found that seniors exhibited dorsolateral prefrontal cortex (DLPFC) over-activation with lower memory loads despite equivalent performance accuracy across age groups. In contrast, with the highest memory load, older adults were significantly less accurate and showed less DLPFC activation compared to their younger counterparts. Likewise, at the behavioral level, we found that the middle-old group performed as good as younger adults, until a point when cognitive demand was high, and performance dropped to the level of older adults.

The counter argument would be that aging affects cognition gradually, with a linear and steady decline in cognitive abilities as people get older. A longitudinal design and/or the inclusion of more cohort groups, especially one between our young (aged 20–31) and middle-old (aged 59–71) group would provide further points to the function and therefore a more comprehensive study of the developmental trajectory of aging cognition. However, although the inclusion of a limited number of age groups remains a limitation of the current study, our results still show a degree of “non-linearity” – as measured cross-sectionally – in the effect of aging on cognitive performance and dispersion. The middle old group performed better than the old-old group on a number of tasks and show a young-equivalent dispersion index. The fact that the age-gap between the middle-old and the young group is much larger than the age gap between the middle-old and the old-old group argues in favor of this non-linearity, and is consistent with other studies (Robbins et al., 1998). Moreover, our cohorts were selected based on previous longitudinal and cohort studies showing age-related changes are almost non-existent before age 60 (Schaie, 1996; Zelinski and Burnight, 1997; Hultsch et al., 1998).

### Dispersion Is Associated With Poor Performance

The interpretation discussed in the previous section that the distinct cognitive profile exhibited by the middle-old group is in fact reflecting differences in recruitment of cognitive resources across age groups receives further support when considering the variability within a person across tasks, namely dispersion. Our results show a general trend which is consistent with previous studies: although this only approached statistical significance, the old-old group exhibited a higher dispersion index compared to both the young and the middle-old group (see **Figure 2** and results), while also performing significantly worse than the younger counterparts (Hultsch et al., 2002; Stuss et al., 2003; Rapp et al., 2005; Hilborn et al., 2009; Halliday et al., 2018). Although lack of statistically significant difference between groups in terms of dispersion index limit the strength of our conclusions, we believe the current pattern of data is still informative. For example, we found that the higher the variability across executive measures, the worse the overall performance in the old-old group. In contrast, level of dispersion was not related to performance in the middle-old group, which show a young-equivalent dispersion index. In this group, the index of cognitive reserve was a better predictor of overall performance.

The association between dispersion and cognitive decline is in line with theories of cognitive aging that suggest a reduction in cognitive control and inhibition of irrelevant cognitive processing (Hasher and Zacks, 1988). However, and most importantly, we showed that dispersion, an index of intra-individual variability across tasks, is not a defining feature of cognitive aging, and is, rather, specifically associated with worse performance. Compensatory mechanisms in the middle-old group may have resulted in young-equivalent dispersion as well as better cognitive outcomes compared to old-old adults (Hypothesis A, **Table 1**).

The fact that the middle-old group did not show age-related dispersion while at the same time showed young-equivalent performance on some tasks and old-old-equivalent performance on others (possibly depending on cognitive demand), suggests that the interaction between dispersion and performance can reveal age-related changes even before deficits are observed at the performance level. In other words, despite good performance in the middle-old group, underlying age-related changes are detectable by examining the relationships between reserve and performance. Although conclusions about the developmental trajectories of cognitive aging need to be drawn with caution in cross-sectional studies as this one, results seem to suggest that healthy aging is not characterized by a linear decrease in cognitive function (Hypothesis A, **Table 1**). Differences between age-groups in intra-individual variability across tasks and related cognitive performance are likely to reflect differences in cognitive resource recruitment, as we found specific effect of cognitive reserve factors in older adults who perform better and show less dispersion (see section below).

### Cognitive Reserve Is Beneficial, but Only When We Need/Can Use It

Together with intelligence and curiosity, education is thought to be a proxy of cognitive reserve, which refers to the ability to flexibly and efficiently use available brain resources (Stern, 2002). Noticeably, the middle-old group was the only group benefitting significantly from extra cognitive reserve provided by education, while concurrently showing less dispersion and better performance (in line with Hypothesis A, **Table 1**). Notably, in those tasks where education had an impact on performance (there was a positive correlation between educational level and cognitive outcome), the (negative) relationship between dispersion index and performance was negligible. This was the case for a number of measures in the middle-old group, and also for the old-old group for one measure (TMT A). It may be that the relatively low cognitive demand associated with TMTA (often considered an index of simple speed of processing, Salthouse, 2011) may have prolonged the beneficial effect of education on performance on the old-old group, specifically for this task but not others.

Our results are in line with a cognitive model of successful aging such as the SOC model (Baltes and Baltes, 1990). According to this model, variability across individuals in terms of how ‘successfully’ they age may depend on resources available as well as engagement of compensatory strategies (Li et al., 2001; Lang et al., 2002; Amieva et al., 2014; Silagi et al., 2015). Accordingly, it has been shown that increasing cognitive reserve through education resulted in greater compensatory potential (Scarmeas et al., 2003).

These results are in line with a recent longitudinal analysis reporting education as a key factor determining cognitive decline in healthy aging, even more so than chronological age itself (Passos et al., 2015), until a point of advanced aging when the beneficial effect vanishes (Paulo et al., 2011). Likewise, cognitive reserve (and related compensatory effects) seems to play a role only when cognitive demand is “sufficiently” high. For Word Span 4, there is no significant relationship between performance and either education or dispersion index for any of the age group, although ceiling effects here should prevent us drawing any conclusions. However, when task demand increases (Word Span 5 and 7), there is a positive effect of education on performance and no association with dispersion in the middle-old group. In contrast, in the old-old group, higher dispersion index is associated with worse performance. Notably, although not statistically significant, there is a trend showing an impact of reserve for the oldest group in overall performance, which is not present for young adults. It may be that, despite a general beneficial effect of reserve in cognitive aging, the dispersion index may play a more important role than reserve in characterizing cognition in more advanced aging (e.g., Paulo et al., 2011). Another possibility may be that variability between individuals in our age-groups may have contributed to alter age-related effects when comparing different cohorts, as the old-old group may include “middle-old-group-like” adults. Longitudinal studies could specifically address this question.

### Conclusion, Limitations, and Further Directions

We showed evidence that the study of the dispersion index in cognitive aging can provide a useful and powerful behavioral measure of age-related differences in cognitive deployment of resources. We found that the association between dispersion, aging and performance does not fit all age groups indiscriminately and cannot be predicted solely based on the developmental stage (Hypothesis A, **Table 1**). Specifically, greater dispersion in advanced healthy aging is associated with poor performance, possibly reflecting reduction in cognitive control (Hasher and Zacks, 1988). Non-significant differences in dispersion index between age-groups limit the strength of our conclusions. However, we showed a non-linear trend which has implications for future studies, especially cross-sectional studies, which should be aware of the differences among older sub-group populations.

We acknowledge that there may be the risk for an over-representation of working-memory measures in the present study (Digit Span backward and Word Span task). However, working-memory is one of the major components involved in age-related decline (e.g., Hasher and Zacks, 1988; Hedden and Gabrieli, 2004) and previous evidence has demonstrated that it contributes heavily to compensatory mechanisms in counteracting age-related losses (e.g., Borella et al., 2010; Karbach and Verhaeghen, 2018), more so than other executive processes. These studies would argue for a special role of working-memory in the study of cognitive aging. Additionally, whether the inclusion of more working-memory measures may have affected our results seems unlikely. For example, our results show a significant relationship between dispersion index – as computed – and TMTB/A for the old-old group, where TMTB/A is very much a switching and updating task rather than a working memory task, thus suggesting a significant dispersion-performance link despite the weight of WM measures in the computed dispersion index.

Although some inferences and analogies can be drawn with regards to cognitive re-organization and age-related changes in resource recruitment, further studies will need to combine behavioral analyses with neuroimaging techniques, to investigate cognitive aging and concurrent changes in neuromodulation. Moreover, as a cross-sectional study, these results are open to the usual threats to validity: potentially, cohort-effects may have led to overestimation of the impact of age on group differences (Cozby, 2009). By limiting the age-range to a maximum of 15 years and controlling for factors such as gender and educational level, an attempt was made to minimize any cohort-effect. Further studies should aim to investigate dispersion in relation to performance longitudinally, as this may reveal pattern of cognitive aging which can be identified from early adolescence (Gow et al., 2012). Additionally, larger sample size may reveal stronger and more reliable associations across performance, dispersion index and factors contributing to cognitive reserve.

## Conclusion

Cognition might undergo some changes to either cope with or as an effect of normal aging. Future studies should aim to clarify whether and how cognitive re-organization in senescence can inform our understanding of age-related changes in neuromodulation. If a link exists between cognitive dispersion and age-related changes in neural modulation, then the next challenge would be to design paradigms which are sensible to small differences across individuals to predict and counteract severe cognitive aging. Factors such as education may contribute to prolonged performance through the employment of extra cognitive resources. Future work should investigate this relation further through neuroimaging and aim to identify additional enhancement factors to increase cognitive reserve. This would also include development of new actions and training to facilitate processes of re-organization and optimization, in order to improve the quality of healthy aging for future generations.

## Author Contributions

The study represents the undergraduate research project of SDF, supervised by CH. SDF collected the data, organized the database, performed the statistical analysis, and wrote the first draft of the manuscript. All authors contributed to the conception and design of the study, manuscript revision, and read and approved the submitted version.

## Conflict of Interest Statement

The authors declare that the research was conducted in the absence of any commercial or financial relationships that could be construed as a potential conflict of interest.
